# The Effectiveness of Age-Specific Isolation Policies on Epidemics of Influenza A (H1N1) in a Large City in Central South China

**DOI:** 10.1371/journal.pone.0132588

**Published:** 2015-07-10

**Authors:** Ruchun Liu, Ross Ka-kit Leung, Tianmu Chen, Xixing Zhang, Faming Chen, Shuilian Chen, Jin Zhao

**Affiliations:** 1 Office for Disease Control and Emergency Response, Changsha Center for Disease Control and Prevention, Changsha, The People’s Republic of China; 2 Stanley Ho Centre for Emerging Infectious Diseases, The Chinese University of Hong Kong, Shatin, New Territories, Hong Kong SAR, The People’s Republic of China; The University of Tokyo, JAPAN

## Abstract

During the early stage of a pandemic, isolation is the most effective means of controlling transmission. However, the effectiveness of age-specific isolation policies is not clear; especially little information is available concerning their effectiveness in China. Epidemiological and serological survey data in the city of Changsha were employed to estimate key model parameters. The average infectious period (date of recovery – date of symptom onset) of influenza A (H1N1) was 5.2 days. Of all infected persons, 45.93% were asymptomatic. The basic reproduction number of the influenza A (H1N1) pandemic was 1.82. Based on the natural history of influenza A (H1N1), we built an extended susceptible-exposed-infectious/asymptomatic-removed model, taking age groups: 0–5, 6–14, 15–24, 25–59, and ≥60 years into consideration for isolation. Without interventions, the total attack rates (TARs) in each age group were 42.73%, 41.95%, 20.51%, 45.03%, and 37.49%, respectively. Although the isolation of 25–59 years-old persons was the most effective, the TAR of individuals of aged 0–5 and 6–14 could not be reduced. Paradoxically, isolating individuals ≥60 year olds was not predicted to be an effective way of reducing the TAR in this group but isolating the age-group 25–59 did, which implies inter-age-group transmission from the latter to the former is significant. Isolating multiple age groups increased effectiveness. The most effective combined isolation target groups were of 6–14 + 25–59 year olds, 6–14 + 15–24 + 25–59 year olds, and 0–5 + 6–14 + 25–59 + ≥60 year olds. The last of these isolation schemas reduced the TAR of the total population from 39.64% to 0.006%, which was exceptionally close to the effectiveness of isolating all five age groups (TAR = 0.004%).

## Introduction

Because influenza viruses undergo frequent genetic mutations and RNA fragment recombination [1], they can easily cause epidemics or pandemics [2,3] and pose a significant threat to human health. Strategies for the global prevention and control of influenza include both pharmaceutical and non-pharmaceutical interventions. The former mainly refers to antiviral drugs and vaccine interventions, whereas the latter mainly refers to isolation, quarantine, the improvement of personal hygiene behaviors, actions that increase social distance (such as school closures, the cancellation of collective activities, and the avoidance of crowded places), travel restrictions, and related interventions [4]. During the early stage of a pandemic, a vaccine is usually absent because it must be produced, which generally takes at least half a year. Accordingly, isolation becomes the most important intervention for controlling the transmission of early-stage pandemic diseases.

Very little data of epidemics without interventions is available, a high proportion of cases can be asymptomatic, and there are difficulties in evaluating the effectiveness of prevention and control strategies. Mathematical modeling can play a complementary role in the design and evaluation of influenza control strategies [5–8]. Assessment of the effectiveness of epidemic control by isolation of specific age groups can guide resource allocation and formulation of strategies to target groups. In this study, we used an influenza A (H1N1) epidemic in the city of Changsha, China. Changsha is the capital of the Hunan province of central southern China and has a population of 6,149,468 people as of 2009. This study serves as an example of building a dynamic model of an influenza epidemic in a large urban population with no interventions. Data related to an epidemic, data on three influenza A (H1N1) outbreaks, and data from a serological survey in Changsha were collected to estimate the key model parameters. Based on these estimates, we established a dynamic model that incorporated isolation procedures. This model was then used to evaluate the effectiveness of isolating different age groups.

## Materials and Methods

### Ethics Statement

The data was obtained from the Chinese Information System for Diseases Control and Prevention and field epidemiological survey. This data included information on influenza cases and the individuals enrolled in our serosurvey. Written informed consent was given by participants (or next of kin/guardians in the case of children) for their clinical information. This study was approved by the Medical Ethics Committee of the Changsha Center for Disease Control and Prevention (CDC).

### Data collection

#### Database of the influenza A (H1N1) epidemic in Changsha

An influenza A (H1N1) epidemic ravaged Changsha city from May 22, 2009 to March 13, 2010. The data was collected from the Chinese Information System for Diseases Control and Prevention. The diagnostic criteria was in accordance with the *Diagnosis and Treatment for H1N1 influenza A* (first to third editions, 2009) [9]. Imported cases are defined as cases of the disease that occurred in patients who had recently travelled to Changsha from other locations. Our study included all imported case information from the early stage of the epidemic, which was used to calculate the basic reproduction number (*R*_0_).

#### Database of the small-scale influenza A (H1N1) outbreaks in school

We collected the information on all outbreaks of influenza A (H1N1) that occurred in school after March 13, 2010 in Changsha city, China. The data was used to calculate the infectious period of influenza A (H1N1) and to estimate the parameter *γ* (recovery rate of the symptomatic). The outbreaks were handled in accordance with the *Chinese Influenza Surveillance Program* (2010^th^ edition) [10] and the *Guidelines for Control of Influenza Outbreak* (2012^th^ edition) [11], as published by the National Health and Family Planning Commission of the People’s Republic of China. Throat swab specimens were collected from patients in order to analyze influenza A (H1N1) virus RNA using polymerase chain reaction (PCR). The diagnostic criteria for influenza was taken from the *Guidelines for Influenza Diagnosis and Treatment* (2011^th^ edition), which was also published by the National Health and Family Planning Commission of the People’s Republic of China [12].

#### Serosurvey database of the influenza A (H1N1) in Changsha

During January, March, and August 2010, we conducted three serosurveys targeting residents of urban Changsha. In each survey, 1,500 subjects were selected by multi-stage random sampling at the city, county/district, neighborhood, household, and individuals levels. In total, 4,500 subjects were selected from five age groups (0 to 5 years, 6 to 15 years, 16 to 24 years, 25 to 59 years, and 60 years and older) with approximately 900 people in each age group. For adults aged ≥18 years informed consent was obtained directly and for those aged <18 years informed consent was provided by both the minors themselves and their parents or legal guardians. Using a standard questionnaire, an individual investigation was conducted for each identified subject: 5 mL of blood was collected from each subject aged over 6 years and approximately 2–3 mL was collected from children aged below 6 years. Serum was obtained via centrifugation. Samples were tested using the hemagglutination inhibition (HI) assay. HI antibody titers with ≥1:40 dilutions were considered to be positive.

### Dynamic model

**Fig 1:**
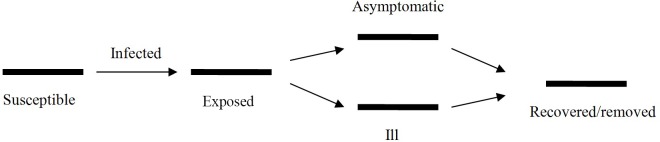
Natural history of influenza A (H1N1).

Our model was based on the natural history of influenza A (H1N1). A susceptible individual is infected by sufficient contact with an ill or asymptomatic person. At first, a newly infected person has an incubation or latent disease status, after which he or she may either become asymptomatic or symptomatic. Asymptomatic individuals then recover with immunity to further infection. Symptomatic individuals either recover with immunity or die of the disease. Because asymptomatic cases are not identified, only symptomatic cases are recorded as cases of influenza in the databases. Accordingly, individuals progress through the phases of 1) being susceptible to influenza, 2) being exposed to influenza (latent or incubation/presymptomatic state), 3) being infectious (including both symptomatic and asymptomatic cases), and 4) being removed from the population at risk of infection (Fig 1). Because an influenza pandemic generally lasts 1 to 1.5 years [13–15], the influences of birth and natural death in the targeted population were omitted from our mathematical model. Hence, a closed susceptible-exposed-infectious/asymptomatic-removed (SEIAR) model is suitable for simulating an influenza A (H1N1) epidemic [16]. In our model, we divided the entire population into five age groups (0–5, 6–14, 15–24, 25–59 and ≥60 years old), which were represented by the numbers 1 to 5, respectively. The model is expressed by the following differential equations:

$$\left\{ \begin{array}{l} dS_{i}\;/\;dt=-\lambda_{i}S_{i}\\ dE_{i}\;/\;dt=\lambda_{i}S_{i}-p_{i}\omega_{A}E_{i}-(1-p_{i})\omega_{I}E_{i}\\ dI_{i}\;/\;dt=(1-p_{i})\omega_{I}E_{i}-\gamma I_{i}\\ dA_{i}\;/\;dt=p_{i}\omega_{A}E_{i}-\gamma '\;A_{i}\\ dR_{i}\;/\;dt=\gamma '\;A_{i}+\gamma I_{i} \end{array}\right.$$ (1)

Where $\lambda_{i}=\sum_{j=1}^{5}\beta_{ji}S_{i}(\kappa \;A_{j}+I_{j})$, *i* = 1, 2, 3, 4, 5.

In these equations, we have susceptible (*S*), exposed (*E*), symptomatic (*I*), asymptomatic (*A*), and removed (*R*) individuals. *dS/dt*, *dE/dt*, *dI/dt*, *dA/dt*, and *dR/dt* refer to the changing rates of the *S*, *E*, *I*, *A*, and *R* populations, respectively. *β*, *ω*_*A*_, *ω*_*I*_, *γ*, *γ*', *κ*, and *p* refer to the transmission relative rate, the rate of transition into an asymptomatic state (*A*), the rate of transition into a symptomatic state (*I*), the rate of recovery from an asymptomatic state, the rate of recovery or death from a symptomatic state, the relative risk of transmission by an asymptomatic individual versus by a symptomatic individual, and the proportion of asymptomatic infections, respectively. *λ* is the force of infection and *λS* is the transfer rates from the *S* to *E* population. *β*_*ji*_ refers to the transmission relative rate from age group *j* to *i*, for example, *β*_23_ refers to the transmission relative rate from age group 6–14 to age group 15–24. The *β* parameters of different age groups are shown in Table 1.

**Table 1:** Transmission rate *β* among different age groups when *R*_0_ = 1.82.

Age groups (years)	0–5	6–14	15–24	25–59	60–
0–5	3.1841×10^−15^	9.8991×10^−7^	4.4281×10^−8^	9.0370×10^−11^	9.6323×10^−10^
6–14		1.2724×10^−8^	2.7278×10^−10^	2.3250×10^−10^	2.0694×10^−8^
15–24			2.9642×10^−8^	8.6064×10^−9^	1.2200×10^−8^
25–59				1.6047×10^−7^	7.9288×10^−8^
≥60					3.0439×10^−7^

### Case isolation

In practice, milder influenza cases were requested to stay home. Dedicated staff visited the affected individuals to ensure adherence, hygiene, and proper disinfection. More severe cases were hospitalized to provide sufficient health care and were isolated. Patients in isolation were discharged three days after the day on which their fevers abated and one day after the day on which they had become free of symptoms. In the case isolation model, none of the *I*-to-*S* paths was viable as a means of transmission. For example, if the 0–5 age group was isolated, cases in this age group could not transmit the disease to the *S* compartment of any age group. Nevertheless, individuals in compartment *S* could become infected via the asymptomatic-susceptible routes of transmission. In this case, the force of infection could be expressed by the following equation:

$$\lambda_{i}=\beta_{1i}S_{i}\kappa \;A_{1}+\sum_{j=2}^{5}\beta_{ji}S_{i}(\kappa \;A_{j}+I_{j}),\;i=1,2,3,4,5.$$

### Estimation of parameters

Estimated model parameters and baseline data are shown in Table 2. Studies have revealed that the incubation period of influenza is 1 to 7 days (mean 1.9 days), the mean latent period is 1.2 days, the mean infection period of asymptomatic patients is 4.2 days (asymptomatic individuals are also contagious [8,12,17]), and the infectivity of asymptomatic individuals is about half that of symptomatic individuals [8,17]. Therefore, we set *ω*_*I*_ = 0.5263, *ω*_*A*_ = 0.8333, *γ*' = 0.2439, and *κ* = 0.5.

**Table 2:** Parameter definitions and values.

Parameter	Description	Unit	Value	Range	Method
*R*_0_	Basic reproduction number	1	1.82	1-	Analysis of epidemic data
*β*	Person-to-person contact rate	day^-1^	See Table 1	0–1	Curve fitting
*k*	Relative transmissibility rate of asymptomatic individuals (versus symptomatic individuals)	1	0.5	0–1	References [8, 12, 14]
*ω*_*I*_	Incubation relative rate	day^-1^	0.5263	0.1429–1	References [8, 12, 14]
*ω*_*A*_	Latent relative rate	day^-1^	0.8333	0.1429–1	References [8, 12, 14]
*p*	Proportion of asymptomatic individuals	1	0.4593	0–1	Analysis of serosurvey data
*γ*	Recovery rate of the symptomatic	day^-1^	0.1923	0.0833–1	Analysis of outbreak data
*γ'*	Recovery rate of the asymptomatic	day^-1^	0.2439	0.0714–1	References [8, 12, 14]

In our study, the three small-scale outbreaks in school formed a baseline source of data that was used to calculate the mean infectious period of influenza cases (providing an estimate of the parameter *γ*). Furthermore, the serological survey database was used to estimate the proportion of the population who were infected with influenza A (H1N1) asymptomatically (providing a value for *p*).

Using all the information on imported influenza cases from the early 2009 Changsha influenza epidemic (dating from May 26 to July 27 of that year), Eq 2 was employed to calculate the basic reproduction number (*R*_0_) for each case [18–20]. The basic reproduction number is defined as the expected number of secondary infections that result from introducing a single infected individual into an otherwise susceptible population [21]. For the case of a single infected compartment, *R*_0_ is simply the product of the infection rate and the mean duration of the infection [22]. Therefore, *R*_0_ can be expressed as follows:

$$R_{0}=\frac{h}{l\times \gamma }$$ (2)

Where, *h* denotes the secondary cases during the non-intervention period, *l* denotes the number of days preceding the intervention, and 1/*γ* denotes the mean natural infectious period.

Thereafter, we calculated the average *R*_0_ of all imported cases and used it to estimate infectivity of imported influenza A (H1N1) in Changsha. Based on this *R*_0_, a typical epidemic was simulated using the SEIAR model that had been built by Chen *et al*. [23] and Arino *et al*. [16]. Subsequently, we fit model 1 with this typical epidemic to calculate each *β*_*ji*_.

### Simulation methods

Berkeley Madonna 8.3.18 (University of California at Berkeley, Berkeley, USA) and Microsoft Office Excel 2003 (Microsoft, Redmond, USA) software were employed for model simulation and figure development, respectively. Differential equation fitting was performed using the Runge-Kutta method of order 4 with the tolerance set at 0.001. While the curve fit is in progress, Berkeley Madonna displays the root-mean-square deviation between the data and the best fit that has been run so far [24].

## Results

### The population sizes of Changsha city

The population sizes of age groups 0–5, 6–14, 15–24, 25–59, and ≥60 years old were 459,660 (7.47%), 576,426 (9.37%), 931,852 (15.15%), 3,353,461 (54.53%), 828,069 (13.47%) respectively.

### Timing of the influenza A (H1N1) epidemic in Changsha

On May 26, 2009, the first case of influenza A (H1N1) was confirmed in Changsha, followed by a number of imported cases. Starting in late July, there was local transmission of influenza. Subsequently, an outbreak and large-scale transmission occurred in Changsha. On January 21, 2010, the Changsha influenza A (H1N1) epidemic ended; thereafter, only sporadic cases were reported. A total of 6,908 cases were reported during the entire epidemic. Contact tracing reveals that the imported influenza cases dated from May 26 to July 27 of that year (See S1 File).

### Infectious period of influenza A (H1N1)

**Fig 2:**
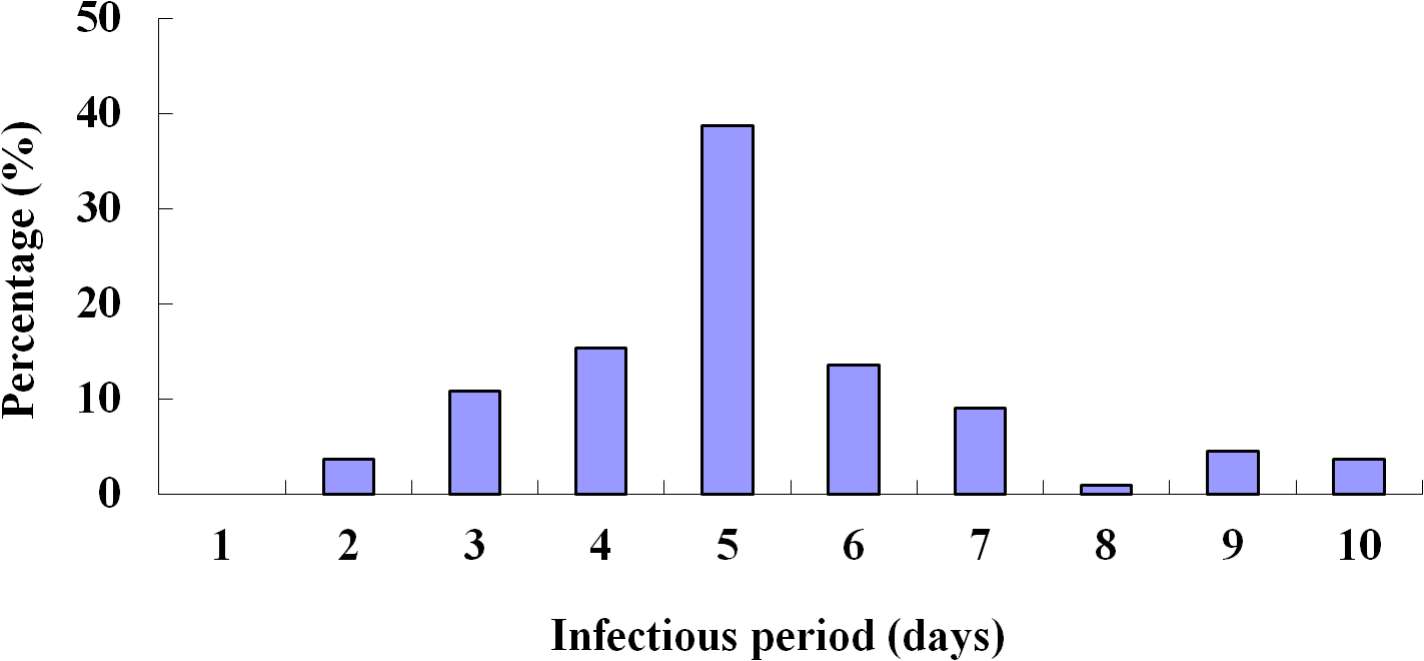
Infectious periods of influenza A (H1N1) cases (n = 111).

All the outbreak data since January 21, 2010 were retrieved, accounting for three outbreaks with 111 cases in total (See S2 File). Among these cases, the shortest course of disease was 2 days, the longest one was 10 days, and the mean was 5.2 days (standard deviation: 1.8 days) (Fig 2). The removal rate of symptomatic individuals was therefore estimated as *γ* = 0.1923.

### Results of the serosurvey

The serosurvey data revealed that 3,650 of the 4,500 respondents had not been vaccinated against influenza A (H1N1), among whom 971 persons were positive for influenza A (H1N1) antibodies, constituting a positivity rate of 25.28% (See S3 File). Among these 971 unvaccinated persons, 446 did not show any influenza-related symptoms between May 2009 and August 2010. Therefore, these were asymptomatic individuals, translating to the proportion of asymptomatic individuals being 45.93%, i.e. *p* = 0.4593. The survey also indicated that the total attack rate (TAR) was 25.28% × (1–0.4593) = 13.67%. The values of *p* in the age groups 0–5, 6–14, 15–24, 25–59, and ≥60 were 0.4511, 0.4177, 0.5026, 0.4459, and 0.5039, respectively (Table 3).

**Table 3:** Value of parameter *p* in different age groups.

Age groups (years)	No. of symptomatic	No. of asymptomatic	*p* (%)
First time			
0–5	54	37	40.66
6–14	76	35	31.53
15–24	37	31	45.59
25–59	20	17	45.95
≥60	10	20	66.67
Second time			
0–5	45	43	48.86
6–14	43	24	35.82
15–24	31	22	41.51
25–59	43	25	36.76
≥60	30	19	38.78
Third time			
0–5	47	40	45.98
6–14	19	40	67.80
15–24	28	44	61.11
25–59	19	24	55.81
≥60	23	25	52.08
Sum of the three times			
0–5	146	120	45.11
6–14	138	99	41.77
15–24	96	97	50.26
25–59	82	66	44.59
≥60	63	64	50.39
Total	525	446	45.93

### *R*_0_ of influenza A (H1N1)

Twenty imported cases of influenza A (H1N1) were identified in Changsha during the early stage of 2009 swine-origin H1N1 influenza A epidemic. Before the local CDC intervened, 305 people had close contact with these 20 individuals in a day on average, resulting in seven secondary cases. This can be translated as 0.35 secondary case per index case during the non-intervention period. Thus *h* = 0.35, *l* = 1, on average. According to the definition of *R*_0_ [21–22] and Eq 2, *R*_0_ = 0.35/1×5.2 = 1.82.

### SEIAR model simulations for influenza A (H1N1) with no intervention

**Fig 3:**
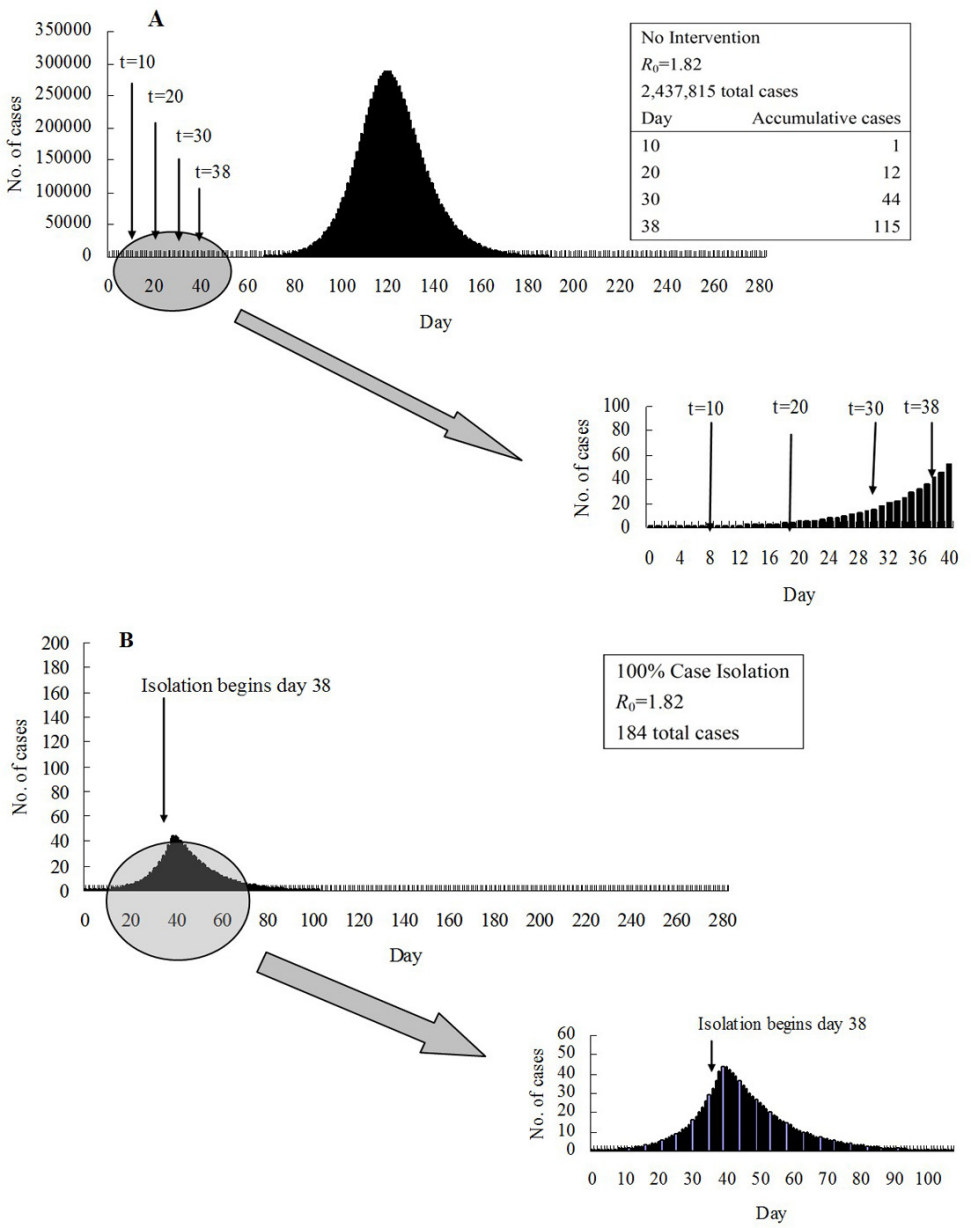
Model simulations. (A) A typical large simulated influenza epidemic with no intervention and *R*_0_ = 1.82. Also shown are the main intervention initiation times that were considered and the number of cases at those intervention times. (B) A typical simulated influenza epidemic which is contained using 100% case isolation initiated 38 days after the first case, when *R*_0_ = 1.82.

We simulated the epidemic curve with *R*_0_ = 1.82 and the *β* parameter of each age group was then obtained by fitting the curve and model (1) (see Table 1). Under these circumstances, the simulated total number of cases in the whole population was 2,437,815, amounting to a total attack rate (TAR) of 39.64% (Fig 3-A), and the simulated TARs of age groups 0–5, 6–14, 15–24, 25–59, and ≥60 were 42.73%, 41.95%, 20.51%, 45.03%, and 37.49%, respectively. Note that these TARs are higher than the TARs reported in the previous section because they simulate a scenario in which no intervention was performed. In contrast, the serosurvey results were obtained following an epidemic that was combatted using vaccination and isolation procedures.

### Effectiveness of isolation

**Fig 4:**
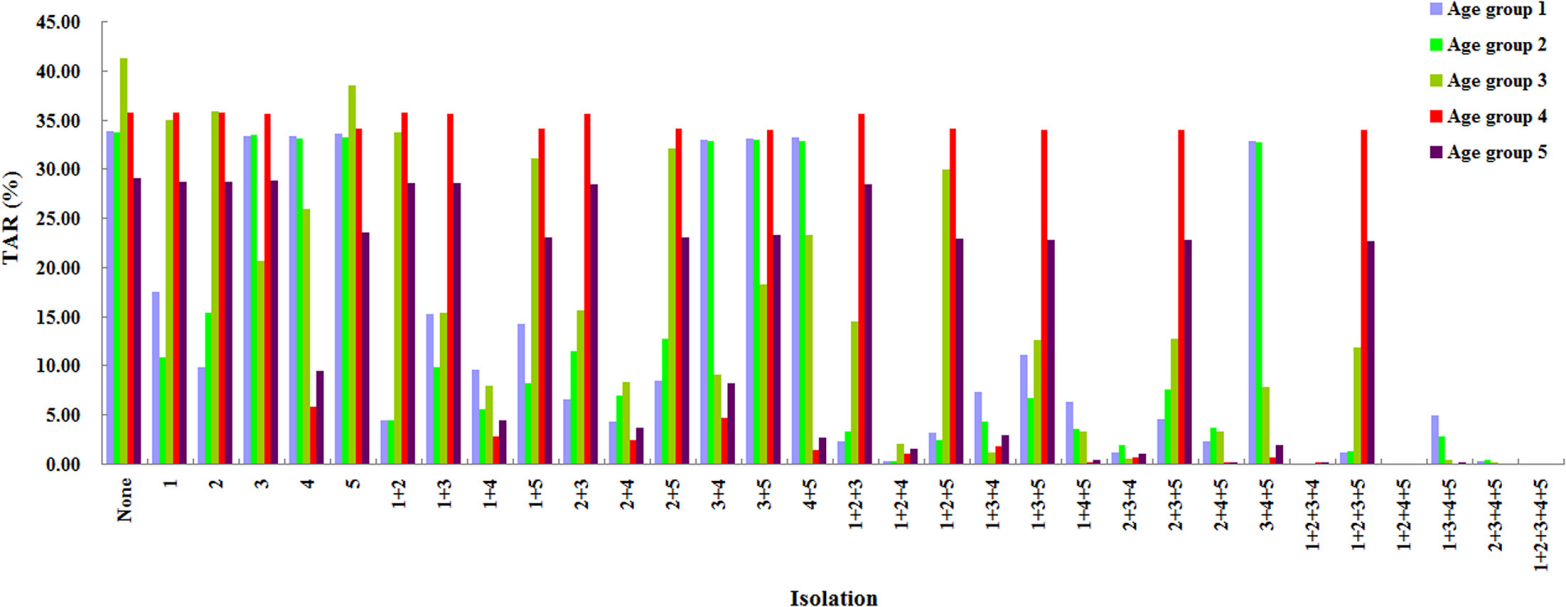
Effectiveness of different combinations of age-group specific isolation.

In our model, isolation began on day 38, when the number of accumulative cases was 115 (Fig 3-A). As shown in Fig 3-B, if all cases were isolated, the number of cases decreased quickly, reaching 0 at day 106 with a total occurrence of only 184 cases. We evaluated the performance of different combinations of age-specific isolation (Fig 4). Groups 1 and 2 had a similar response; isolating either one was predicted to reduce the TAR of the other. The group 3 TAR was intractable, remaining at least as high as about 10% when only two age-groups were isolated. To suppress the group 3 TAR to a low value, group 4 had to be isolated, plus any two of groups 1, 2 and 3. The TAR of group 4 could only be suppressed by isolating the group themselves; and isolating group 4 usually could bring down the TAR of the others. In particular, the group 5 TAR could only be reduced effectively when group 4 was isolated. In contrast, the TAR of either group 1 or 2 could not be suppressed by isolating group 4.

Isolating any single age group predicted high TARs in the non-isolated groups. When only two age groups were isolated, the isolation of age groups 2 and 4 (persons aged 6–14 and 25–59 years old) attained relatively low TARs in all age groups. Among the policies in which three age groups were isolated, the most effective strategy was the isolation of age groups 2, 3, and 4 (persons aged 6–59 years old). The most effective isolation strategy involving four age groups (age groups 1, 2, 4, 5) had similar performance as that involving all five age groups.

## Discussion

In this study of the 2009 influenza A (H1N1) epidemic, the average duration from symptom onset to recovery was estimated to be 5.2 days and the basic reproduction number was estimated to be 1.82, this agreed in general with the previous values [8,17,25–27]. We estimated that the overall proportion of asymptomatic infection (*p*) was 45.93% and found that this proportion was age group specific; implying that using the overall *p* for different age groups may introduce large errors.

Although satisfactory outcomes were not obtained by isolating any single age group, as expected the isolation of persons 25–59 years old was predicted to be the most effective, likely because it comprised the largest portion in population, reducing the TAR of the total population from 39.64% to 14.49%. Nevertheless, targeting the control at working adults is also likely to be the most economically expensive, due to that age group being the most numerous, this means that significant amount of targeting costs may have to be spent.

Isolating more than three age groups was predicted to have marginal benefit. Isolating a single age group can risk an uncontrolled TAR in other non-targeted groups. But isolation of the two age groups may strike a proper balance between available resources and public health benefits. Based on the actual effectiveness of the control measures that were used in the Changsha epidemic (TAR: 13.67%), according to our simulation results, the TAR could be controlled to as low as 0.004% if all age groups were isolated; and only about four percent if only groups 2 and 4 or 1 and 4 were isolated. Consequently, there is room for improvement for the prevention and control of epidemic in Changsha.

Of note, our study did not take into account factors such as climate and population flows, which could affect the modeling results to some extent. Yet, the key parameters of our model were estimated based on data related to an epidemic, data on three influenza A (H1N1) outbreaks, and data from a serological survey in Changsha, which are therefore well suited to simulate the influenza epidemic in Changsha. During future epidemics, our mathematical simulation results could be used as a reference to improve the efficiency of prevention and control measures.

## Supporting Information

S1 File Timing of the influenza A (H1N1) epidemic in Changsha. (XLS)

S2 File Infectious periods of 111 cases. (XLS)

S3 File Results of three serosurveys. (XLS)
